# Sounds facilitate visual motion discrimination via the enhancement of late occipital visual representations

**DOI:** 10.1016/j.neuroimage.2017.01.010

**Published:** 2017-03-01

**Authors:** Stephanie J. Kayser, Marios G. Philiastides, Christoph Kayser

**Affiliations:** Institute of Neuroscience and Psychology, University of Glasgow, Glasgow, UK

**Keywords:** Audio-visual, EEG, Single trial decoding, Sensory decision making, Motion discrimination

## Abstract

Sensory discriminations, such as judgements about visual motion, often benefit from multisensory evidence. Despite many reports of enhanced brain activity during multisensory conditions, it remains unclear which dynamic processes implement the multisensory benefit for an upcoming decision in the human brain. Specifically, it remains difficult to attribute perceptual benefits to specific processes, such as early sensory encoding, the transformation of sensory representations into a motor response, or to more unspecific processes such as attention. We combined an audio-visual motion discrimination task with the single-trial mapping of dynamic sensory representations in EEG activity to localize when and where multisensory congruency facilitates perceptual accuracy. Our results show that a congruent sound facilitates the encoding of motion direction in occipital sensory - as opposed to parieto-frontal - cortices, and facilitates later - as opposed to early (i.e. below 100 ms) - sensory activations. This multisensory enhancement was visible as an earlier rise of motion-sensitive activity in middle-occipital regions about 350 ms from stimulus onset, which reflected the better discriminability of motion direction from brain activity and correlated with the perceptual benefit provided by congruent multisensory information. This supports a hierarchical model of multisensory integration in which the enhancement of relevant sensory cortical representations is transformed into a more accurate choice.

## Highlights

• Feature specific multisensory integration occurs in sensory not amodal cortex.
• Feature specific integration occurs late, i.e. around 350 ms post stimulus onset.
• Acoustic and visual representations interact in occipital motion regions.

## 1 Introduction

Multisensory integration can improve perceptual performance across a wide range of tasks. While there is an emerging consensus that the underlying neural correlates likely involve multiple stages of the sensory decision making pathways, it remains a challenge to uncover the dynamic processes that implement the multisensory benefit for an upcoming decision in the human brain (Bizley et al., 2016, Kayser and Shams, 2015, Rohe and Noppeney, 2014, Rohe and Noppeney, 2016). For example, many studies have shown that judgements about visual motion can be influenced by simultaneous sounds (Alais and Burr, 2004, Beer and Roder, 2004, Lewis and Noppeney, 2010, Schmiedchen et al., 2012) or vestibular information (Fetsch et al., 2010, Gu et al., 2008), even so when the multisensory stimulus is not directly task relevant (Gleiss and Kayser, 2014b, Kim et al., 2012, Sekuler et al., 1997). In particular, congruent multisensory evidence enhances visual motion discrimination performance over incongruent multisensory information (Meyer and Wuerger, 2001, Meyer et al., 2005, Soto-Faraco et al., 2003, Soto-Faraco et al., 2002). Yet, it remains difficult to attribute these perceptual benefits to specific neural processes, such as the encoding of visual motion in occipital cortices, the transformation of sensory representations into a motor response in parieto-frontal regions, or to more unspecific changes in sensory-response gain such as attentional effects (Beer and Roder, 2004, Bizley et al., 2016, Lewis and Noppeney, 2010, Talsma et al., 2010).

Electrophysiological studies in monkeys have illustrated in great detail how neural populations in visual motion regions, such as the Medial Superior Temporal Area (MSTd), combine directional information from the visual and vestibular senses to yield a more precise and reliable estimate of the perceived motion direction (Fetsch et al., 2013, Fetsch et al., 2012, Gu et al., 2008). These neurons weigh the two sensory inputs in proportion to each senses reliability, in a similar way as the behavioural benefits arise from the combination of visual and vestibular information (Angelaki et al., 2009, Fetsch et al., 2009). While this could be taken to suggest that multisensory benefits for visual motion discrimination in the human brain are similarly arising from an enhancement of the encoding of visual motion in occipital regions, we still have a limited understanding of when and where the underlying neural processes operate. While fMRI studies support a central role of visual motion cortex in mediating multisensory benefits (Alink et al., 2008, Lewis and Noppeney, 2010, Scheef et al., 2009), studies on other tasks such as spatial localization have provided a more nuanced picture, one in which multiple occipital and parietal regions contribute distinctively to multisensory integration (Rohe and Noppeney, 2014, Rohe and Noppeney, 2016). For example, while studies using planar motion have implied the hMT complex (but see (Baumann and Greenlee, 2007)), a study on motion in depth has pointed to a role of area V3A (Ogawa and Macaluso, 2013) and regions within the IPS (Guipponi et al., 2013). Given the frequent focus on mapping activations rather than sensory representations (Kriegeskorte et al., 2006), and given that many prior studies have relied on the relatively slow fMRI-BOLD response, these studies do not provide a detailed understanding of where and when during a trial perceptually relevant multisensory benefits emerge and are transformed into perceptual benefits on a single trial basis (Bizley et al., 2016, Zhang et al., 2016).

Exploiting the temporal resolution of EEG or MEG, a few studies have investigated the neural mechanisms of audio-visual interactions in the context of motion perception. Studies focusing on auditory cortical activity have shown that the congruency of visual information can affect auditory brain activity already at latencies of around 100 ms (Stekelenburg and Vroomen, 2009, Zvyagintsev et al., 2009) while occipital evoked responses were affected by cross-modal attention around 200 ms post-stimulus onset (Beer and Roder, 2005), and occipital oscillatory activity was affected by Audio-visual motion congruency already around 100 ms (Gleiss and Kayser, 2014b). However, these EEG/MEG studies also focused on mapping generic activations rather than mapping sensory representations, and the use of trial-averaged activity made it difficult to link neural mechanisms to the perceptual single trial benefits.

We hence reasoned that EEG-based neuroimaging combined with the single trial mapping of task-relevant sensory representations could provide important insights about the neural processes mediating the multisensory enhancement of motion discrimination. In particular we exploited an information-mapping approach, in which we used single trial decoding to select EEG activations that are relevant to the subjects’ behaviour and task, rather than studying single electrode ERPs. Our specific aims were to test whether acoustic information enhances the quality of early or later visual representations in occipital cortex, or manifests mostly in decision-related processes in parieto-frontal regions and immediately before the response. To this end we combined a standard motion discrimination task with single-trial EEG analysis to map the relevant dynamic representations of visual motion direction. We then asked when in time during a trial EEG activations carrying the task-relevant visual information were modulated by multisensory congruency and whether these activations localized to sensory cortices, or fronto-parietal association regions.

To better understand the potential role of attention-related processes in multisensory perception we also extracted parietal alpha activity and related this to the observed behavioural benefits and the neural encoding processes. The power of parietal alpha has been linked to visual spatial attention and the excitability of visual cortices (Romei et al., 2010, Thut et al., 2006, VanRullen, 2016), with higher (lower) power being potentially indicative of reduced (increased) attentional focus. As previous work has suggested that alpha power can change with multisensory congruency (Gleiss and Kayser, 2014b), we sought to replicate this effect, and to test whether a change in alpha band activity contributes to multisensory perceptual benefits at the single trial level, for example by modulating the contribution of sensory information to perceptual choice.

## 2 Materials and methods

Data were obtained from 18 healthy adult participants (8 males; mean age of 21.3 years) following written informed consent and briefing about the purpose of the study. All had self-reported normal hearing and vision, declared no previous history of neurological disorders and were right-handed (Oldfield, 1971).The study was conducted in accordance with the Declaration of Helsinki and was approved by the local ethics committee (College of Science and Engineering, University of Glasgow).

### 2.1 Experimental design and stimulus material

**Fig. 1:**
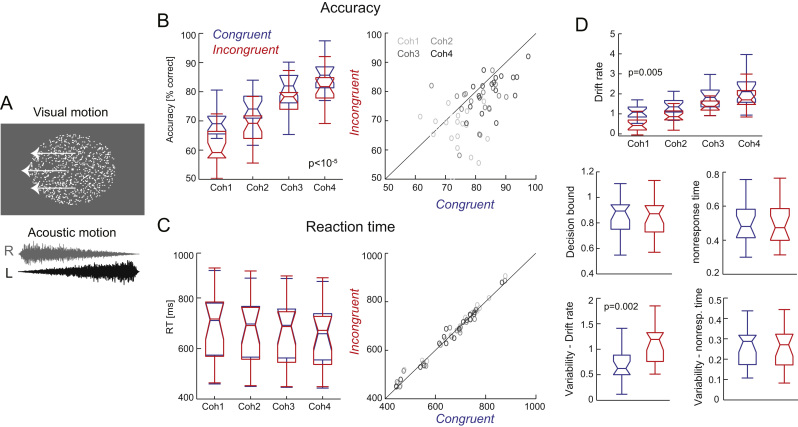
Experimental paradigm and behavioural data. A) Subjects performed a speeded visual motion discrimination task (left- or right-wards). Random dot motion was presented at four coherence levels (coh 1–4) titrated around each participant's perceptual threshold. Visual stimuli were accompanied by acoustic motion implemented by changing levels of sound intensity between ears, either moving in the same (congruent) or opposite direction (incongruent) as the visual stimulus. B) Perceptual accuracy increased significantly with motion coherence and was significantly higher during congruent trials. C) Reaction times did not change significantly with coherence or congruency. D) Parameters derived from drift-diffusion models fit to behavioural data, with significant congruency effects in drift rates and their variability. Variability = Inter-trial variability. Boxplots: medians and percentiles across participants (n=18).

Subjects discriminated the direction (left- or rightwards) of visual motion presented in a random dot display (Fig. 1A). Stimuli were presented following the onset of a fixation dot (0.7-1.1 s uniform delay) and lasted 1.2 s. Individual trials were separated by 1.5-2 s intervals. Random dot patterns (1400 dots, white, presented on a neutral grey screen, 4 cd/m2 background luminance) were centred on the fixation spot and covered 15° of visual angle (with the centre 1° devoid of dots). Individual dots were 0.2° large, moved at 6°/s in a random direction and 8% of dots were randomly replaced after each frame (16 ms). A small percentage of dots moved coherently in the same direction (left or right). This fraction could take four different values titrated around each participant's perceptual threshold. These thresholds (around 71% correct responses) were determined in a separate session using three interleaved 2-down 1-up staircases. During the actual experiment the coherence level was adapted (in steps of 1%) over epochs of 35 trials to adjust for changes in performance over time (Gleiss and Kayser, 2014b). Across subjects coherence thresholds were comparable (10.7±1.4%; mean±s.e.m) and varied on average by 1.3% over time (subject averaged standard deviation). The four coherence values used during the experiment were defined as [0.55, 0.85, 1.15, 1.45] times the subject specific threshold. As a result, the range of motion coherence spanned from challenging to relatively easy, as confirmed by the variation in average performance from about 60% to nearly 90% correct across conditions (Fig. 1B). Visual stimuli were presented on a 21" Hansol 2100A CRT monitor at a refresh rate of 85 Hz. These visual stimuli were accompanied by a dynamic acoustic stimulus mimicking motion in either the same or the opposite direction as the visual motion. Hence, the acoustic direction cue was either congruent or incongruent with the visual direction. Sounds were composed from white noise (at 44.1 kHz sampling rate) whose amplitude was linearly modulated from 0 to the maximal level in opposite directions on left and right ears during the 1.2 s stimulus period. This change in inter-aural level difference induces the percept of continuous acoustic motion (Meyer and Wuerger, 2001, Moore, 2003). Sounds were presented with a peak amplitude of 65 dB(A) SPL r.m.s. level; on- and offsets were cosine ramped (8 ms). The reliability of the onset timing of sounds and random dot patterns was verified using an oscilloscope. Both stimuli reliably appeared within one refresh cycle of the screen (~11 ms).

The different conditions (left-, rightwards motion), four visual coherence levels, and two Audio-visual congruencies were pseudo-randomized and balanced across trials. Trials were presented in blocks of 240 and each subject completed 1200 trials, resulting in 150 trials per condition of interest (four coherence levels x two levels of congruency). Subjects were instructed ‘to discriminate the direction of visual motion and to respond as quickly and accurately as possible and to ensure they respond within the stimulus period’ by pressing a left or right arrow key on a keyboard, using the same hand for both keys. To achieve a stable speed-accuracy trade-off subjects performed 40 (or when necessary more) training trials during which they received feedback on accuracy and response time. Negative feedback on response time was given when responding too early (below 0.3 s) or after the stimulus disappeared (later than 1.2 s).

### 2.2 EEG recordings

Experiments were performed in a dark and electrically shielded room. Acoustic stimuli were presented binaurally using a Sennheiser headphone and stimulus presentation was controlled from Matlab (Mathworks) using routines from the Psychophysics toolbox (Brainard, 1997). Sound levels were calibrated using a sound level meter (Model 2250; Bruel&Kjær, Denmark). EEG signals were continuously recorded using an active 64 channel BioSemi system (BioSemi, B.V., The Netherlands) using Ag-AgCl electrodes mounted on an elastic cap according to the 10/20 system. Four additional electrodes were placed near the outer canthi and below the eyes to obtain the electro-oculogram (EOG). Electrode offsets were kept below 25 mV. Data were acquired at a sampling rate of 500Hz using a low pass filter of 208Hz.

### 2.3 General data analysis

Data analysis was carried out offline with MATLAB (The MathWorks Inc., Natick, MA), using the FieldTrip toolbox (Oostenveld et al., 2011) and custom written routines similar to previous work (Kayser et al., 2016). Data from different blocks were pre-processed separately by band-pass filtering (1 Hz-70 Hz), re-sampling to 150Hz and de-noising using ICA. ICA components reflecting eye movement induced artefacts, highly localized muscle activity or poor electrode contacts were identified and removed following definitions provided in the literature (Hipp and Siegel, 2013, O'Beirne and Patuzzi, 1999). To determine periods contaminated by blinks or eye movements we computed horizontal, vertical and radial EOG signals (Keren et al., 2010) and rejected trials in which potential eye movements were detected based on a threshold of 3 standard deviations above mean of the high-pass filtered EOGs, or during which the peak amplitude on any electrode exceeded ±120 μV. We also excluded trials in which reaction times where shorter than 0.3 s or longer than the trial (1.2 s). Together this led to the rejection of 9.2±3% of trials (mean±s.e.m). For subsequent analysis the EEG signals were referenced to the common average reference.

### 2.4 Fitting drift diffusion models

We fit the behavioural data (accuracy, reaction times) with a drift-diffusion model for sensory decision making (Ratcliff et al., 2009, Ratcliff et al., 2016). We used a fitting procedure based on partial differential equation describing the diffusion process, as implemented in the fast-dm toolbox using the Kolmogorov-Smirnov procedure (Voss and Voss, 2007). We obtained three model parameters related to the width of the interval between the start of the process and the decision threshold (termed ‘decision bound’ – A), the influence of the stimulus on the diffusion process (‘drift rate’ – k), and the duration of all extra-decisional parts of the response time (‘nonresponse time’ – t0). The drift rate was allowed to vary across conditions (congruency and visual coherence), while the residual time and the bound were assumed to be independent of coherence but were allowed to vary with congruency. We thereby assumed that the decision criterion and processes not related to the decision making process (peripheral sensory processing, motor latencies) are not affected by the coherence of the visual stimulus, while all three parameters were included to potentially explain differences in behavioural performance with multisensory congruency. Parameters relating to inter-trial variability of nonresponse times and drift-rates were left free to vary across congruency conditions. We also assumed that the starting point and the speed of execution of responses did not differ between the two choice options. These assumptions seem justified given that median reaction times did not differ between choices (0.657±0.032 and 0.656±0.030 mean±s.e.m. across subjects for left and right buttons, sign-test p=0.48, Z=0.7), nor did the fraction of correct responses (73.7±1.8 and 74.2±1.3% correct, p=0.81, Z=0.23).

### 2.5 EEG single trial discriminant analysis

We used multivariate linear discriminant analysis to localize EEG activations sensitive to EEG activity reflecting the task-relevant visual information (motion direction) or the subject's choice at the single trial level. We used a regularized linear discriminant analysis (Blankertz et al., 2011, Parra et al., 2005) to identify a projection of the multidimensional EEG data, x(t), that maximally discriminated between the two conditions of interest (motion direction, choice), across all coherence levels and regardless of Audio-visual congruency. Each projection was defined by a projection vector, w, which describes a one dimensional combination of the EEG data, Y:

(1) $$Y\left(t\right)=\sum_{i}w_{i}x_{i}\left(t\right)+c$$

with *i* summing over all channels, and a constant *c*. The regularization parameter was optimized in preliminary tests using cross-validation and kept fixed for all subsequent analyses. The discriminant analysis was applied to the EEG activity in 80ms sliding windows. We searched for discriminant components sensitive to visual motion direction in the data aligned to stimulus onset and aligned to the response, and for discriminant components sensitive to choice in the data aligned to response. Classification performance was quantified using the area under the receiver operator characteristic (Az) based on 6-fold cross validation. Given potentially unequal trial numbers for each condition, we repeated the discriminant analysis 100-times using a random subset of 80% of the available trials for each condition, averaging the resulting Az and projection vectors. We derived scalp topographies for each discriminant component by estimating the corresponding forward model, defined as the normalized correlation between the discriminant component and the EEG activity (Parra et al., 2005).

**Fig. 2:**
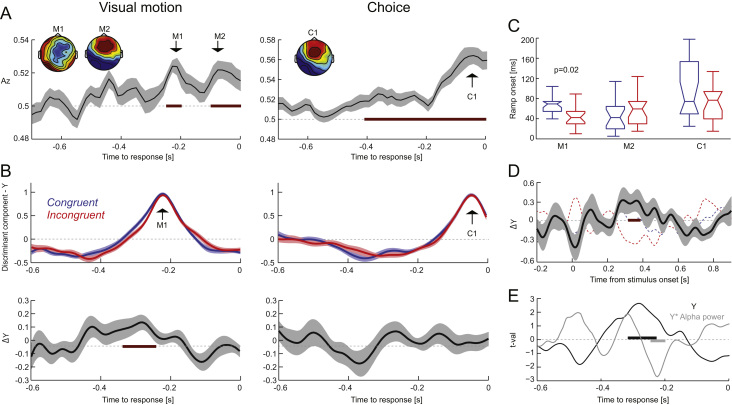
Audio-visual congruency enhances visual motion representations. Single trial linear discriminant analysis was used to extract EEG activations sensitive to the direction of visual motion (left in panels A,B) and to single trial choice (right). A) Discriminant performance (Az: area under the receiver operator characteristic) in data aligned to response. Time epochs with significant performance are indicated in red (at least p<0.01). B) Upper panels: Projection of single trial discriminant components, Y, extracted at time points of interest in the motion or choice discriminants (M1; C1). For a definition of the component activation see Eq. (1). These are shown separately for congruent and incongruent trials, normalized for effects of visual coherence and only for correct trials (see main text for results pertaining to all trials). Lower panels: Statistical contrast for a congruency effect (significant for the visual motion component M1; -0.34 s to -0.25 s; p<10^-5^; not in the choice component, C1). C) Single subject ramp onset times differed significantly between congruent and incongruent trials for the motion component M1 (p=0.02), but not for the motion component M2 or the choice component C1. D) Statistical contrast for a congruency effect in the discriminant component M1 when aligned to stimulus onset (significant from 0.31 to 0.37 s; p=0.001). E) Single trial modelling of choice revealed a significant influence of visual motion direction (p<10^−5^) and of the discriminant component (black; -0.31 s to -0.23 s, p<0.001). Including alpha power into the model furthermore revealed a significant interaction of alpha power with the discriminant component (gray; -0.24 s to -0.20 s, p<0.001). Lines and shaded regions indicate means and standard errors across participants (n=18). Boxplots indicate medians and quartiles. Δ: Congruent – incongruent.

The discriminant activity provides a sensitive and aggregate representation of the underlying task relevant activity (Kayser et al., 2016, Parra et al., 2005, Philiastides et al., 2014). In particular, Y(t) can be exploited as a measure of the single trial sensory evidence (or choice-selective signal), as larger values (either positive or negative) correspond to a better separability of the two conditions of interest. We exploited this to investigate the temporal evolution of the relevant discriminant components by obtaining single trial projections of the discriminant activity by applying the weights extracted at time points of interest (t_peak_) to all trials and time points. Previous work suggests that the underlying signals exhibit a ramping behaviour, whereby they slowly rise prior to t_peak_ (O'Connell et al., 2012, Philiastides et al., 2014). Indeed, we found this to be the case for both visual motion and choice discriminants (Fig. 2B). We compared the strength of the sensory (or choice) evidence in these discriminant components by comparing their amplitude (ignoring the difference in sign arising from the two motion / choice directions) between congruent and incongruent trials, after normalizing out effects of coherence. We repeated this analysis twice, once using all trials in order to be able to direct compare neural and behavioural parameters, and once using only trials with correct performance to rule out potential confounds of accuracy.

To extract an index of when during the trial the evidence reflected by each discriminant component started to rise we computed ‘ramp onset’ times based on the trial averaged single subject data. These onset times were defined as the first time point at which the temporal cumulative sum of Y(t) (in the time range of 250 ms prior to t_peak_) crossed zero from negative to positive. Ramp onset times were defined as the difference between the times of threshold crossing to the time point 250 ms prior to t_peak_, and hence were positive by construction. We note that the precise value of this onset time is ambiguous, as it depends on the threshold and the time window chosen for analysis. However, within and between subject comparisons of conditions are meaningful.

We tested the relevance of the discriminant component for subject's behaviour at the single trial level using logistic regression. The regression model predicted choice based on the task-relevant variable (motion direction), the discriminant activation Y, and in a separate model the interaction of Y with alpha power.

### 2.6 Time frequency analysis

**Fig. 3:**
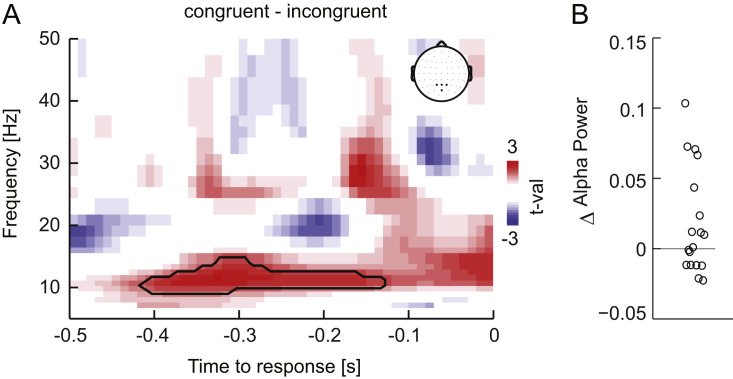
Congruency effect in oscillatory activity. A) Congruency difference in oscillatory power over parieto-occipital electrodes (see inset) with a significant effect in the alpha band (8-14 Hz) between -0.4 s and -0.12 s (p=0.03). B) Change in alpha power for each participant. Power was expressed as ratio of stimulus to baseline periods. Δ: Congruent – incongruent.

Time frequency representations of the oscillatory power were obtained using wavelet analysis in FieldTrip. Frequencies ranged from 4 Hz to 80 Hz, in steps of 1 Hz below 16 Hz and steps of 2 Hz above, using a 5 Hz wavelet width. Trial-averaged representations were baseline normalized to a pre-trial period (-0.5 to -0.1 s before stimulus onset) and were expressed as ratio of stimulus to baseline periods. Given potentially unequal trial numbers, we computed the condition difference in normalized power by choosing a random subset of 80% of the available trials per condition, averaging the normalized differences across 100 repeats. We applied this analysis to pre-selected occipito-parietal electrodes of interest (PO3, PO4, Pz, POz), averaging the power difference across electrodes within each subject. These electrodes were selected based on the prominence of alpha effects around these locations in previous literature (Gleiss and Kayser, 2014a, Gleiss and Kayser, 2014b, Romei et al., 2012, Romei et al., 2008). For further analysis we extracted the single trial baseline-normalized alpha power in a specific time-frequency window of interest derived from the group-level analysis of the congruency effect (Fig. 3A; 9-13 Hz; -0.36 s to -0.28 s).

### 2.7 Source analysis

**Fig. 4:**
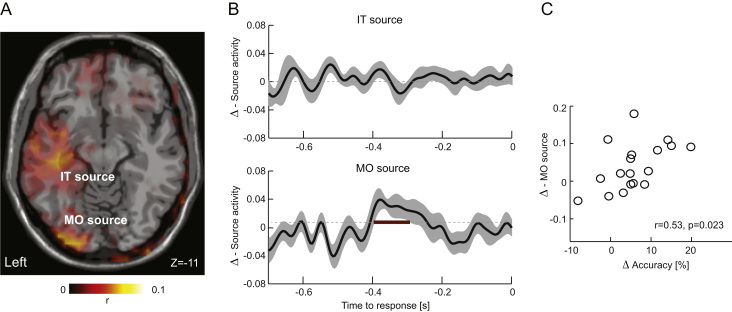
Source analysis of the motion discriminant component M1. A) Group-level correlation map (z-scored; median value) between the discriminant and source activity. This revealed two clusters, one in middle occipital regions (MO) and one in the inferotemporal lobe (IT). Image is in neurological convention. B) Congruency difference in the source activity extracted from these two locations. A significant congruency effect was found only for the occipital source (red line; -0.39 s to -0.28 s; p<10^−5^). C) Across-subject correlation of the congruency effect (Δ) in source activity (averaged between -0.38 s and -0.34 s) and the behavioural accuracy effect. Lines and shaded regions indicate means and standard errors across participants (n=18). Δ: Congruent – incongruent.

To obtain an estimate of the brain regions generating the discriminant component activations of interest we performed a source localization analysis. We first obtained single trial source signals of the response-aligned data using a linear constrained minimum variance beamformer in Fieldtrip (7% normalization, using the covariance matrix obtained from -0.7 to -0.1 s prior to response). A standardized head model based on the average template brain of the Montreal Neurological Institute was used as single subject MRI data were not available. Lead-fields were computed using a 3D grid with 6 mm spacing. We then computed the correlation of single voxel signals with the linear discriminant signal, Y(t), over trials at the single subject level. This is analogous to obtaining the forward scalp distribution via the correlation of sensor activity and discriminant activity (Haufe et al., 2014, Parra et al., 2005). Correlation volumes were z-transformed and we computed the median correlation across subjects. We further analysed the activity at two source locations of interest, by extracting the single-trial source activity at two local peaks of the correlation maps (Fig. 4A).

### 2.8 Statistical analyses

The analysis of behavioural data was based on the Scheirer-Ray-Hare non-parametric two-way ANOVA. Correlations were based on Spearman's rank correlation and bootstrap confidence intervals (95% level) were calculated using the robust correlation toolbox (Pernet et al., 2012). Significance testing of discriminant performance (Az), of congruency effects in discriminant activity, and of differences in oscillatory power at the group-level were based on a cluster-based permutation procedure, which shuffled condition labels and corrected for multiple comparisons along time (and frequency) (Maris and Oostenveld, 2007, Nichols and Holmes, 2002) (detailed parameters: 2000 iterations; clustering bins with abs(t)>1.5, or with Az above the 95% percentile of the distribution across bins; minimal cluster size of at least 4 neighbours; computing the cluster-mass within each cluster; performing a two-sided test at p<0.05 on the clustered data). Where necessary, single subject contrasts were obtained first using t-statistics. For the logistic regression model we derived group-level t-values based on single subject regression betas. We provide exact p values where possible, but values below 10^−5^ are abbreviated as such.

## 3 Results

### 3.1 Behavioural results

Subjects performed a motion discrimination task based on a visual random dot display. They were instructed to respond as accurately and fast as possible (Fig. 1A). In each trial the visual stimulus was accompanied by a sound, which provided an acoustic motion cue either moving in the same or opposite direction as the visual display. As expected, response accuracy significantly improved with the coherence of visual motion (four levels; χ^2^(3)=77, p=<10^−5^, Fig. 1B). Accuracy was also significantly higher during congruent compared to incongruent trials (χ^2^(1)=12, p=0.0004), and there was no interaction between these factors (χ^2^(3)=0.2, p=0.96). Reaction times decreased with coherence, but neither the effects of coherence (χ^2^(3)=4.3, p=0.22; Fig. 1C) nor of congruency (χ^2^(1)=0.01, p=0.91) were significant; there was also no interaction (χ^2^(3)=0.01, p=0.99). Median reaction times varied between 0.44 and 0.82s across subjects, with an overall median of 0.66 s. To further corroborate the lack of an effect of congruency on reaction times we compared, for each subject and coherence, the shape of the reaction time distribution between congruencies using Kolmogorov-Smirnov tests. Across the 4×18 tests there were only three comparisons that reached an uncorrected p<0.05, but when accounting for multiple comparisons there was no significant effect (Benjamini & Hochberg FDR procedure at p<0.05). The scatter plots in Fig. 1B,C illustrate the multisensory benefit for accuracy in the absence of significant a change in reaction times.

### 3.2 Drift diffusion models predict faster accumulation during congruent trials

We fit the behavioural data with a diffusion model for sensory decision making, testing the effect of Audio-visual congruency on drift rates, decision bounds, and nonresponse times. Across subjects drift rates increased significantly with motion coherence (Fig. 1D; χ^2^(3)=12, p=0.005) and were significantly higher during congruent compared to incongruent trials (χ^2^(1)=14, p=0.0001); there was no interaction (χ^2^(3)=3.5, p=0.39). We did not find significant effects of congruency on decision bounds (Wilcoxon test: Z(17)=-0.8, p=0.37) and nonresponse times (Z(17)=-1.1, p=0.26). We also analyzed the inter-trial variability of the drift rate and the nonresponse times. This revealed no significant effect for the nonresponse time (Fig. 1D; Z(17)=-0.4, p=0.67), but a significantly higher variability of the drift rate in the incongruent condition (Z(17)=-3.3, p=0.0021). Given that increases in drift rate generally predict decreases in reaction times, which we did not observe at the group level, we analyzed the decision bound and nonresponse times in more detail. Across subjects congruency effects in these parameters were significantly anti-correlated (r=-0.67, p=0.002, CI [-0.82 -0.38]), suggesting that in addition to a consistent change in the accumulation process multisensory congruency also had heterogeneous influences on other aspects of the sensory decision process. Nevertheless, these modelling results suggest that the most consistent influence of congruent multisensory information arises from an enhancement of the temporal accumulation of visual evidence, embodied by the drift rate of the diffusion model. This conclusion is also consistent with predictions made by a previous study, which suggested that sensory accumulation in multisensory conditions is based on a combination of drift rates of the two unisensory stimuli, and is largest in congruent multisensory environments (Drugowitsch et al., 2014). We hence expected to see a change in the EEG signatures of visual representations with multisensory congruency.

### 3.3 Extracting EEG signatures of sensory encoding and choice

Our goal was to localize EEG activations sensitive to the direction of visual motion or to the subsequent choice, and to probe whether and when these are affected by multisensory congruency. To this end we applied linear discriminant analysis to single trial data. As reaction times varied between participants we searched for motion-sensitive components in the data aligned to both stimulus onset and to response. Discriminant performance for extracting motion sensitive components was not significant in the onset-aligned data, but in the data aligned to response (Fig. 2A; randomization statistics with FWE p<0.01 along time): discriminant performance was significant in two time epochs (M1: -0.25 to -0.2 s, T_sum_=2.0, p<0.01; M2: -0.1 s to 0 s, T_sum_ =9.0, p<10^-5^). The fact that motion selective discriminant components were significant only in the response-aligned data suggests that these components are probably associated more with late and choice-relevant processes rather than early sensory activations. Discriminant analysis for choice revealed one significant time epoch (C1: -0.42 s to 0 s, T_sum_=10.1, p<10^-5^). The scalp projections of these three discriminant components (at their peak times) are shown in Fig. 2A. We next asked whether multisensory congruency influences the sensory or choice evidence reflected by these discriminant components.

### 3.4 Multisensory congruency enhances visual motion evidence

To analyse the time course of these discriminant components we obtained single trial projections of the respective discriminant activations. These are shown in Fig. 2B (left for the visual motion component derived at t_peak_=-0.23 s, ‘M1’; right for the choice component derived at t_peak_ =-0.08 s, ‘C1’), normalized for the effect of visual coherence, and only for correct responses to rule out influences of performance on these components. As expected, these discriminant components exhibited a ramp-like behaviour over a period of about 200 ms before t_peak_ (O'Connell et al., 2012, Philiastides et al., 2014). Importantly, when contrasting congruent and incongruent conditions we found a significant difference for the motion component M1 (cluster-based randomization statistics, FWE p<0.01; T_sum_=32, p<10^-5^): motion evidence was significantly stronger and started to rise earlier during congruent trials in a window between -0.34 s and -0.25 s. Importantly, this congruency effect at the same latencies also persisted when we analyzed all correct and incorrect trials together (T_sum_=28, p<10^-5^).

To further confirm this multisensory enhancement we extracted ramp onset times for these rising discriminant signals, defined based on all (i.e. correct and incorrect) trials. Ramp onset times differed significantly between congruencies, confirming an earlier rise of motion representations during congruent over incongruent trials (Fig. 2C; median values: congruent 70ms, incongruent 42 ms; Wilcoxon test: Z(17)=2.3, p=0.02). Not surprisingly, the stronger discriminant activity during congruent trials also resulted a better discriminability of visual motion direction based on the EEG activity (Az averaged over the significant time window and coherence levels, congruent: 0.55±0.006; incongruent: 0.53±0.004, mean±s.e.m.; sign-test p=0.0075).

To directly test whether the single trial evidence provided by this discriminant component (M1) was predictive of subject's choice we entered the discriminant activation and the actual motion direction into a logistic regression of choice, after normalizing Y within each coherence level (Fig. 2E). Not surprisingly, the effect of motion direction was highly significant (t(17)=21, p<10^-5^). More importantly, the effect of discriminant component was significant around a similar time window as the congruency effect (-0.31 s to -0.23 s; T_sum_=36, p<0.001), indicating that this EEG signature has a significant impact on subjects’ responses beyond the influence of the physically visible stimulus. In line with this result we also found that the shift in the ramp onset times was significantly correlated with the change in drift rate predicted by the diffusion model across subjects (r=0.69, p=0.0015, CI [0.31, 0.89]). The shift in ramp onset times was also significantly anti-correlated with the change in the inter-trial variability of the drift rate (r=-0.49, p=0.035, CI [-0.80, -0.01]). As a result, an earlier onset of the motion-sensitive discriminant component in congruent multisensory conditions was associated with a more reliable (in a trial by trial sense) and faster accumulation of sensory evidence in the drift diffusion model fitted to behavioural performance.

To probe whether the enhancement of visual motion representation by multisensory congruency was specific to the motion component M1, we also obtained projections of the motion component M2 (derived at t_peak_ =-0.05 s). There was no significant effect of congruency at any time point in these projections. Furthermore, the ramp onset times extracted from these did not differ between congruencies (Fig. 2C; median values: congruent 42 ms, incongruent 60ms; Wilcoxon test: Z(17)=-1.3, p=0.19). We also did not find a significant effect of congruency on the projections of the choice-sensitive component (C1; t_peak_ =-0.05s; Fig. 2B left for the time course; median ramp onsets: congruent 75ms, incongruent 74ms; Wilcoxon test: Z(17)=0.9, p=0.32). The two later components (M2, C1) seem to index similar processes, given that they emerge around the same time and have similar topographies. Yet, these discriminant components are unlikely to be purely motor-plan related, given that subjects used the same hand for both responses and that EEG cannot discriminate activations related to different fingers of the same hand. Furthermore, the topography does not seem to be consistent with the well-known lateralised motor potential. All in all, this suggests that Audio-visual congruency influences the dynamic evolution of visual motion representations about 300ms prior to the response, but does not specifically enhance later motion sensitive discriminant components or choice selective signals immediately before the response.

To obtain a better understanding of the time point during a trial at which this congruency effect emerges, we obtained single trial projections of the motion component M1 when aligned to stimulus onset (Fig. 2D). To this end we applied the discriminant weights obtained from the response-aligned discriminant analysis to the time series of the onset-aligned data. This revealed a significant congruency effect around 0.31 s to 0.37 s post stimulus onset (T_sum_=13, p=0.001). Together with the response-aligned data (effect around 300 ms pre-response) and the typical reaction times (around 660ms) this suggests that the multisensory EEG signature emerges at latencies intermediate between stimulus onset and response.

### 3.5 Changes in alpha power facilitate sensory encoding benefits

Previous studies have reported changes in parieto-occipital alpha power with multisensory congruency. Given that parietal alpha has been linked to visual spatial attention and the excitability of visual cortices these findings have been interpreted as attentional contributions to multisensory perceptual benefits. Hence we asked whether there was a similar effect of congruency on parietal alpha power in the present data. We computed time-frequency representations in response aligned data and quantified the congruency effect over pre-selected occipito-parietal sensors (Fig. 3A). As expected (Gleiss and Kayser, 2014a, Gleiss and Kayser, 2014b), alpha power was significantly higher during congruent compared to incongruent trials, between -0.4 s and -0.12 s and 8-14 Hz (T_sum_=237.4, p=0.03). However, the distribution of changes in alpha power with congruency was highly variable, and only 10 of 18 participants exhibited higher power during congruent trials (Fig. 3B). To obtain a more specific understanding of whether and how alpha power contributes to shaping subjects’ single trial behaviour, we included an interaction of alpha power with the discriminant component (M1) in the regression of choice. This interaction was significantly negative in a time window of -0.24 s to -0.20 s (Fig. 2E; T_sum_=-16.5, p<0.001), hence subsequent to the peak in the motion evidence reflected by this discriminant component. This suggests that reduced alpha power subsequently reinforces the impact of the encoded motion evidence on behavioural responses during the formation of choice.

### 3.6 Motion sensitive discriminant components localize to visual motion regions

We performed a source localization analysis to obtain a better understanding of the brain regions from which the visual motion sensitive discriminant component (M1) arises. We computed trial by trial correlations between single voxel activity and the discriminant activation at the single subject level at each point during the trial, in analogy to the definition of forward scalp distribution of linear discriminant components (c.f. Methods) (Haufe et al., 2014, Parra et al., 2005). Group-level median correlation maps (extracted at t_peak_ =-0.23 s) revealed two clusters of positive correlations (Fig. 4A). These localized to an inferotemporal source (MNI [-40 -29 -11]; AAL atlas label: Temporal Inf L), and an occipital source (MNI [-29 -94 -11]; AAL atlas label: Occipital Mid L). Given that we observed a significant congruency effect in the discriminant activation both when aligned to response (Fig. 2B), and when aligned to stimulus onset (Fig. 2D), we repeated the source localization analysis using the stimulus-aligned data. This confirmed the same two sources as obtained from the response-aligned analysis. Furthermore, while these maps suggest a left-lateralization of the source correlation, a statistical comparison of group-level correlation values of the left occipital source with the corresponding values extracted from the right hemisphere did not reveal a statistically significant difference (Wilcoxon test; median values 0.078 for left and 0.009 for the right hemispheres; Z=1.9, p=0.058).

To quantify the sensitivity to multisensory congruency of these sources we further analysed the respective single trial signals. Group level statistics for a congruency effect (cluster-based randomization statistics, FWE p<0.01) revealed no effect at the inferotemporal source, but a significant congruency effect at the occipital source, which emerged around the same time as the congruency effect in the discriminant component extracted from the sensor data (-0.39 s to -0.28 s; T_sum_=47, p<10^-5^). Finally, to test whether these source signals were linked to the perceptual benefit we correlated the congruency effects in accuracy (congruent minus incongruent) with the congruency difference in the source activations around the time of the peak differences (averaged in -0.38 s to -0.34 s; Fig. 4C) across subjects. This correlation was significant for the occipital (r=0.53, p=0.023, CI [0.05, 0.86]) but not the inferotemporal source (r=-0.05, p=0.81, CI [-0.53, 0.41]), suggesting that multisensory benefits for the neural representation of visual motion evidence in occipital cortex directly relate to the perceptual benefit.

## 4 Discussion

Our results show that a congruent sound facilitates the encoding of visual motion direction in occipital sensory regions. This was evident as an earlier rise of the visual motion sensitive discriminant component in congruent compared to incongruent trials about 350 ms following stimulus onset, and about 300 ms prior to the response. This earlier emergence of task relevant sensory representations reflected the better discriminability of visual motion direction from brain activity. Furthermore, the respective discriminant activation was significantly predictive of subjects’ single trial choice and the congruency effect in occipital brain activity was predictive of the respective accuracy benefit provided by congruent over incongruent multisensory evidence. Together this reveals the multisensory facilitation of later sensory processing stages in occipital regions that subsequently drive perceptual choice.

### 4.1 Congruent acoustic information enhances occipital sensory representations

The when and where of multisensory integration has been attributed to a wide range of regions in the brain. While older studies had pointed to high level parietal and prefrontal association regions, many studies in the last decade have suggested that multisensory interactions occur already at the earliest cortical or even subcortical stages (Ghazanfar and Schroeder, 2006, Kayser and Logothetis, 2007, Schroeder and Foxe, 2002). In particular, many studies have argued that behaviourally relevant multisensory interactions can occur around primary-like sensory cortices and at very early latencies relative to stimulus onset (Ibrahim et al., 2016, Murray et al., 2016, Schroeder and Foxe, 2005, van Atteveldt et al., 2014). However, recent studies suggest that there may be no generic answer to this question, as multisensory processing likely involves a distributed set of task- and function-specific regions (Bizley et al., 2016, Werner and Noppeney, 2010). In line with this hypothesis, two recent fMRI studies have illustrated how the computational nature of Audio-visual interactions changes from low-level sensory to high-level parietal cortices (Rohe and Noppeney, 2014, Rohe and Noppeney, 2016).

In the context of motion perception both intracranial recordings and functional imaging studies in humans have demonstrated that multisensory information can enhance sensory representations in occipital motion cortex (Alink et al., 2008, Poirier et al., 2005, Sadaghiani et al., 2009). While electrophysiological studies have described the computational rules by which MSTd neurons combine visual and vestibular information in great detail (Angelaki et al., 2009, Fetsch et al., 2013), less is known about the multisensory response properties of the human motion cortex. Some studies have shown that non-visual directional evidence can directly modulate hMT responses (Alink et al., 2012, Baumann and Greenlee, 2007, Bedny et al., 2010, Poirier et al., 2005, Saenz et al., 2008, Scheef et al., 2009, van Kemenade et al., 2014), and one study suggested that perceptual benefits may arise directly from the enhancement of hMT responses (Lewis and Noppeney, 2010). However, it remained unclear whether multisensory activations in motion cortex arise early in time relative to stimulus onset, and hence likely reflect bottom up mechanisms related to the stimulus-driven encoding of sensory information (Kayser and Logothetis, 2007, Schroeder and Foxe, 2002, Werner and Noppeney, 2010). Alternatively, multisensory activations could arise at longer latencies and hence possibly result from top-down feedback mechanisms that relate multisensory information back to early sensory cortices (Nath and Beauchamp, 2011, Vetter et al., 2014).

We here capitalize on the mapping of sensory representations rather than generic response amplitudes in functional imaging data (Kayser et al., 2016, Kriegeskorte et al., 2006, Philiastides et al., 2014). Our approach differs from previous EEG studies in that we did not quantify multisensory effects on individual ERPs, which potentially capture many different neural processes. Rather, we relied on single trial discriminant analysis to select relevant EEG components that carry task-relevant sensory representations, here about the direction of visual motion. Our results corroborate the importance of occipital cortices in mediating the acoustic facilitation of visual motion discrimination. We directly demonstrate that the underlying visual representations are significantly predictive of subjects’ single trial choice, and that their multisensory facilitation is predictive of the accuracy benefit. While the precision of EEG source localization is on the order of a few centimetres (Song et al., 2015), our results nevertheless constrain the origin of the multisensory benefit to occipital sensory representations rather than parieto-frontal regions. Our findings hence support an origin of multisensory encoding benefits within sensory-specific cortices in opposition to domain general and amodal regions (Ghazanfar and Schroeder, 2006, Hanks et al., 2015, Murray et al., 2016, Raposo et al., 2014). At the same time our results also demonstrate an origin within a high-level occipital region, in opposition to primary visual cortices. Our results localize the neural correlates of multisensory enhancement to intermediate epochs of the trial, about 350 ms from stimulus onset and about 300 ms before the response. This contrasts with suggestions of low latency multisensory interactions, such as changes in the N100 amplitude or latency (Giard and Peronnet, 1999, Roa Romero et al., 2015, Stekelenburg and Vroomen, 2007, Stekelenburg and Vroomen, 2009, van Wassenhove et al., 2005, Zvyagintsev et al., 2009) or similar effects with latencies shorter than 100ms from stimulus onset (Giard and Peronnet, 1999, Murray et al., 2004).

We interpret our results as support for a hierarchical model of multisensory integration. In such a model the earliest multisensory effects reflect changes in sensory saliency or expectancy, driven by the synchronous and possibly redundant information arriving to different senses (Kayser et al., 2010, Schroeder and Foxe, 2005, Schroeder et al., 2008, Talsma et al., 2010). Later effects, in contrast, reflect computationally specific mechanisms relating to the combination of feature-specific information which are implemented in the respective sensory cortices carrying the task-relevant representations. These later interactions are shaped by task-demands, the relevance and suitability of each modality for the specific task (Bizley et al., 2016, Kayser and Shams, 2015, Rohe and Noppeney, 2014, Werner and Noppeney, 2010). While the earlier interactions likely emerge automatically and in a bottom-up manner, the later interactions are dependent on feedback from higher association regions, which guide multisensory influences in sensory cortices contingent on task requirements. This task-dependency of multisensory interactions may in part also contribute to differences in the timing and location of the neural correlates of behavioural benefits observed in the literature. A neural origin within motion-sensitive regions in the present study is likely given the task nature (motion direction discrimination) and it is possible that the use of a different visual stimulus (e.g. static stimuli, or speech) or a different task (e.g. shape discrimination, or phosphene detection) could result in neural correlates that emerge at a different latency or in other sensory cortices (Giard and Peronnet, 1999, Romei et al., 2012, Romei et al., 2009, Stekelenburg and Vroomen, 2007, van Wassenhove et al., 2005).

We here used an acoustic motion stimulus created using intensity differences between the ears based on sounds presented via headphones. The use of headphones can induce an apparent spatial mismatch between the acoustic and visual stimuli. This lack of co-localization can reduce the perceptual integration benefit, and may hence influence the observed neural correlates (Beer and Roder, 2004, Frassinetti et al., 2002, Meyer et al., 2005, Rohe and Noppeney, 2016, Soto-Faraco et al., 2002). To complicate matters further, the influence of Audio-visual disparity on behavioural integration itself may be task dependent. Studies on the detection of coherent motion (Meyer et al., 2005) or flashes of dim light (Frassinetti et al., 2002) reported a tolerance of up to 20 degrees of Audio-visual disparity, while studies on stimulus localization in the context of causal inference suggest a more narrow binding window (Kording et al., 2007, Rohe and Noppeney, 2015, Rohe and Noppeney, 2016). As a result, it remains possible that potentially earlier integration effects could be observed under conditions where the apparent spatial discrepancy in the sensory environment, and hence the need for the brain to analyse the causal structure of the environment in great detail, is reduced.

### 4.2 EEG-informed mapping of sensory decision processes

Our interpretation that multisensory information enhances late occipital sensory representations is also in line with studies on purely visual decision making. Several EEG studies have localized correlates of the sensory and evidence accumulation processes driving choice (Ratcliff et al., 2016). Patterns of ramping activity have been observed within sensory and fronto-parietal regions during different tasks (O'Connell et al., 2012, Philiastides et al., 2010, Philiastides and Sajda, 2006, Polania et al., 2014, Tremel and Wheeler, 2015), with some components likely reflecting the accumulation of evidence within sensory cortices (Tremel and Wheeler, 2015). For example, in the context of visual object processing, Philiastides and Sadja identified a late (~300ms) ERP component attributed to lateral occipital cortex, which correlated with the drift rate derived from diffusion models (Philiastides et al., 2006, Philiastides and Sajda, 2006, Philiastides and Sajda, 2007). Similarly, intracranial recordings in animals have shown patterns of ramping activity within motion sensitive cortex (Britten et al., 1996, Shadlen and Kiani, 2013) and multisensory parietal regions (Hanks et al., 2015) that are predictive of the animals choice, which, in a multisensory context, can also carry information about the modality composition of the stimulus (Raposo et al., 2014).

While our source localization results cannot dissect contributions from motion cortex and more lateral occipital regions, the data reinforce the notion of a late but sensory-specific multisensory enhancement. The ramp onset times of the early motion discriminant component changed with multisensory congruency, and this change correlated with the congruency effect in drift rates: an earlier rise of the EEG component was associated with higher and more reliable drift rates. This EEG correlate of evidence accumulation emerged around 350 ms following stimulus onset, and just around the time at which the sensory encoding stage ends and the decision process begins as predicted by the diffusion model: the nonresponse times were around 480 ms (median), and assuming a 100 ms for motor action, this leaves 380 ms for early sensory encoding. The congruency effect in the stimulus-aligned data emerged between 310 ms and 370 ms, hence just prior to the onset of the decision process. The choice selectivity observed in intracranial recordings from visual motion cortex (Britten et al., 1996) and parietal regions (Hanks et al., 2015, Shadlen and Kiani, 2013) usually emerges at latencies of around 50 ms to 200 ms respectively. This is considerably earlier than the choice relevance of the visual motion component that exhibited the multisensory congruency effect in the present study (Fig. 2E). One reason for this difference could be the nature of the different signals. However, a later emergence of the behaviourally-relevant neural multisensory interaction could also reflect the involvement of top-down processes that steer the low-level sensory encoding contingent on task requirements, sensory reliabilities, or other high-level inference processes (Rohe and Noppeney, 2014, Rohe and Noppeney, 2016).

The context sensitivity of multisensory perception predicted by the inference perspective also raises another intriguing question regarding the influence of task and temporal context. A well-known property of decision making is that congruency effects, such as in the Stroop or Eriksen flanker tasks, are stronger following a congruent than following an incongruent trial (Gratton et al., 1992, Mayr and Awh, 2009, Schmidt et al., 2007). While it remains unclear whether the origin of these serial order effects is more on the cognitive (Botvinick et al., 2004, Carter et al., 1998) or sensory side of neural processes (Mayr and Awh, 2009, Schmidt and De Houwer, 2011), multisensory studies have reported similar serial order effects, such as changes in the temporal binding window or a bias in spatial localization estimates (Van der Burg et al., 2013, Van der Burg et al., 2015, Wozny and Shams, 2011b). These are often interpreted in the context of sensory recalibration, as they could arise from a shift in the representation of the encoded sensory likelihoods (Wozny and Shams, 2011a). However, these multisensory effects could possibly also originate from amodal and general decision making processes. Future work is required to disentangle multisensory serial congruency effects from amodal processes and to map these onto their respective neural origins.

### 4.3 Attentional modulation of multisensory processing

Previous work has shown that multisensory integration and attentional selection are deeply intertwined. Attention can facilitate the binding across modalities by amplifying co-occurring objects, but can also reduce the likelihood of integration in complex scenes by limiting the range of objects that are likely to be bound (Beer and Roder, 2004, Beer and Roder, 2005, Macaluso et al., 2016, Talsma et al., 2006, Talsma et al., 2010). We have recently reported that auxiliary multisensory effects, i.e. multisensory benefits arising from stimuli that by themselves do not offer task relevant information, can in part be explained by processes typically associated with visual attention (Gleiss and Kayser, 2014a, Gleiss and Kayser, 2014b). For example, the perceptual accuracy benefit for detecting visual motion in a two interval task correlated with changes in parieto-occipital alpha power (Gleiss and Kayser, 2014b), a prominent marker of visual attention and the related control of visual excitability (Busch and VanRullen, 2010, Romei et al., 2009, Thut et al., 2012, Thut et al., 2006). The present results confirm a group-level increase of parieto-occipital alpha power during congruent trials, which could be interpreted as a requirement for less attentional resources in a congruent environment (Gleiss and Kayser, 2014b). However, single trial modelling revealed a contrasting picture, in which visual sensory representations have a stronger impact on subsequent choice when alpha power is reduced (Fig. 2E). Hence, and not very surprising, on a single trial basis increases in attention seem to be predictive of better performance.

These findings fit well with the hierarchical view of multisensory integration. Previous work has suggested that the role of attention in multisensory perception depends on whether multiple stimuli fit with the assumption of a common origin, a property that is likely shaped not only by spatio-temporal proximity but also the overall likelihood of each experimental condition, e.g. congruency, to occur within a given experimental paradigm (Talsma et al., 2010, Vatakis and Spence, 2007). Following this interpretation sensory information propagates to high level sensory areas in the parietal lobe, which implement the causal inference process (Rohe and Noppeney, 2014, Rohe and Noppeney, 2016). The outcome of this triggers the attentional amplification of the relevant sensory representations in visual cortices at latencies that match the recurrent amplification of sensory representations (Arnal and Giraud, 2012, Philiastides and Sajda, 2007). While our results provide direct evidence for the late enhancement of occipital sensory representations, future work is required to place this into a context of a general multisensory inference process (Deroy et al., 2016, Rohe and Noppeney, 2014).

### 4.4 Conclusion

We used an information-mapping, rather than activation-mapping, approach to investigate the neural correlates of multisensory integration. Using single trial analysis we extracted the task-relevant neural representations and asked when during a trial and where in the brain these are enhanced in a congruent multisensory context. Our results point to sensory-cortical rather than fronto-parietal processes and to activations that emerge relatively late during a trial. These findings support the multisensory nature of sensory cortices and fit well with the notion of a hierarchical organisation of multisensory processing in the brain.
